# Molecular Identification and Pathogenicity of a Chilli Veinal Mottle Virus Isolate from Sichuan, China

**DOI:** 10.3390/ijms27021113

**Published:** 2026-01-22

**Authors:** Cheng Chen, Daihua Lu, Xiaotong Lin, Xueping Zhou, Xiuling Yang

**Affiliations:** 1 Institute of Plant Protection, Sichuan Academy of Agricultural Sciences, Key Laboratory of Integrated Pest Management on Crops in Southwest, Ministry of Agriculture and Rural Affairs, Chengdu 610066, China; zbschencheng@scsaas.cn (C.C.); ludaihua@scsaas.cn (D.L.); 2 State Key Laboratory for Biology of Plant Diseases and Insect Pests, Institute of Plant Protection, Chinese Academy of Agricultural Sciences, Beijing 100193, China; 18860320626@163.com; 3 State Key Laboratory of Rice Biology, Institute of Biotechnology, Zhejiang University, Hangzhou 310058, China

**Keywords:** phylogenetic analysis, recombination analysis, infectious clone, infectivity, NIb

## Abstract

Chilli veinal mottle virus (ChiVMV) is an important potyvirus that poses a serious threat to crop production. In this study, small RNA sequencing and molecular cloning were used to obtain the complete genome sequence of a ChiVMV isolate identified in pepper plants in Sichuan (SC1 isolate). Molecular evolutionary and phylogenetic analysis of SC1 and 35 ChiVMV isolates revealed four clades of ChiVMV isolates. Recombination analysis found 23 recombinant events and 28 recombinants, with the SC1 isolate arising from the recombination of the PK isolate from Pakistan and the YNpe isolate from Yunnan, China. A full-length infectious cDNA clone of ChiVMV was constructed and demonstrated to be infectious in both *Nicotiana benthamiana* and pepper plants. Moreover, a Myc-tag was inserted after NIb, and the derived infectious clone of ChiVMV remained infectious, and NIb-Myc was readily expressed in infected host plants. These reverse genetic tools will promote the study of the function of ChiVMV-encoded proteins, especially the NIb protein, and facilitate basic and translational studies of ChiVMV.

## 1. Introduction

Pepper (*Capsicum* spp.) is a globally significant vegetable crop cultivated for both fresh consumption and dried products as spices [1]. According to FAOSTAT (2023), worldwide pepper cultivation spanned 3.87 million hectares in 2023, yielding approximately 38.31 million tons of fresh pepper fruit and 5.82 million tons of dried pods (FAOSTAT, 2023, https://www.fao.org/faostat/zh/#data, accessed on 15 January 2026). In China, the annual planting area of pepper consistently exceeds two million hectares, accounting for 8–10% of the country’s total vegetable cultivation area [2]. However, pepper production is severely constrained by its susceptibility to numerous pathogens, especially viruses, which reduce yield and fruit quality [3]. It has been reported that at least 166 viruses can infect peppers worldwide, and severe epidemics have been documented to cause yield losses exceeding 80% [4,5].

Chilli veinal mottle virus (ChiVMV; also known as pepper vein-banding mosaic virus or chilli vein-banding mottle virus) is an emerging potyvirus that poses a severe constraint to pepper production [6]. It was first identified in West Malaysia in 1979 [7] and has since been reported in Tanzania [8], China [9], India [10], Thailand [11], and Pakistan [12]. ChiVMV mainly infects pepper (*Capsicum* spp.) and other solanaceous crops such as tobacco, tomato, and eggplant [8,13,14]. ChiVMV is transmitted by mechanical sap inoculation and by several species of aphids in a non-persistent manner [15]. Pepper plants infected by ChiVMV exhibit mottling, mosaic, vein banding, necrotic ringspots, distortion, reduced size on leaves, and diminished size and yield of fruits [16]. Different isolates of ChiVMV exhibit genetic variation, and recombination has been identified as a key factor driving evolution of ChiVMV populations, resulting in divergent symptom severity in their host plants. For example, the Pp3 and Yp7 isolates, collected from different pepper-producing areas, showed different pathogenicity in their host plants [17].

ChiVMV is a positive single-stranded RNA virus with a genome of approximately 9.7 kb. It encodes a single large polyprotein that is proteolytically processed into 11 mature viral proteins: P1, HC-Pro, P3, PIPO, 6K1, CI, 6K2, VPg, NIa, NIb, and CP [18]. These proteins orchestrate critical steps in the viral life cycle, such as protease cleavage, suppression of RNA silencing, virus replication, cell-to-cell movement, and aphid transmission [19]. Among these viral proteins, the nuclear inclusion b (NIb) protein, which serves as the RNA-dependent RNA polymerase (RdRp), is indispensable for potyvirus replication and acts as a central hub for virus–host interactions [20]. NIb recruits a number of host proteins to viral replication complexes (VRCs) and interacts with other viral proteins, such as 6K2, VPg, and HC-Pro, to synergistically promote viral infection [21,22]. NIb also plays a critical role in the suppression of host antiviral immunity [23,24]. Despite its well-established critical functions in other potyviruses, the function of the NIb protein encoded by ChiVMV remains largely uncharacterized.

In this study, ChiVMV was isolated from *Capsicum annuum* plants collected in Sichuan Province, China. The complete genome sequence of this isolate was determined, and its molecular evolution and phylogenetic relationships with other known ChiVMV isolates were analyzed. A full-length infectious cDNA clone of ChiVMV was constructed and demonstrated to be infectious in both *Nicotiana benthamiana* and its natural host, *C. annuum*. Furthermore, a Myc-tag was introduced to the C-terminus of NIb in the infectious clone. The resulting recombinant virus remained pathogenic and stably expressed the NIb-Myc fusion protein in infected plants. The reverse genetic tools developed in this study enable future studies of the function of ChiVMV-encoded proteins and plant-virus interactions.

## 2. Results

### 2.1. Identification of Chilli Veinal Mottle Virus

**Figure 1:**
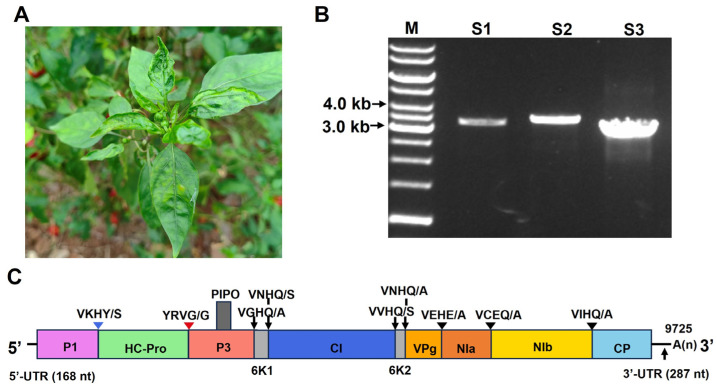
Characterization of ChiVMV in pepper. (**A**) Symptoms observed on ChiVMV-infected pepper plants in Sichuan Province of China. (**B**) Amplification of the full-length ChiVMV genome in three segments (S1, S2, S3). Lane M, GeneRuler 1 kb DNA ladders (Thermo). (**C**) Genomic organization of ChiVMV. The proposed cleavage sites of the polyprotein are denoted by vertical lines, and the corresponding amino acids are shown.

In June 2022, *C. annuum* plants showing leaf crinkle and chlorosis symptoms were observed in Chengdu, Sichuan Province, China (Figure 1A). To identify potential virus(es) present in the diseased *C. annuum* plants, leaf samples were collected and subjected to small RNA deep sequencing. After filtering adapter and low-quality sequences, a total of 13,625,995 clean reads were obtained. The clean reads of 18–26 nt in length were assembled, and 102 contigs related to viruses were generated. BLASTn (version 2.17.0+) analysis against the GenBank database revealed that 79 contigs shared the highest sequence similarities to the genomic sequences of ChiVMV (GenBank accession numbers KC711055.1; MK405594.1; KC711056.1), and 22 contigs had the highest sequence similarities to the genomic sequences of pepper cryptic virus 1 (GenBank accession numbers MH782162.1; MH782163.1). Given its widespread distribution and impact on solanaceous crops and the lack of effective control strategies, ChiVMV was selected for further analysis.

The complete genome of the SC1 isolate of ChiVMV was cloned by RT-PCR, and the sequences were determined and assembled (Figure 1B). The SC1 isolate has a genome length of 9725 nt and contains a large open reading frame (ORF) encoding a polyprotein of 3089 amino acids. This ORF is flanked by a 5′ untranslated region (UTR) of 168 nt and a 3′ UTR of 287 nt. Similarly to the genome organization of other potyviruses, the polyprotein of the SC1 isolate is presumed to be proteolytically processed into 11 mature proteins, including P1, HC-Pro, P3, PIPO, 6K1, CI, 6K2, VPg, NIa, NIb, and CP (Figure 1C).

### 2.2. Comparative Genome and Phylogenetic Analysis

**Figure 2:**
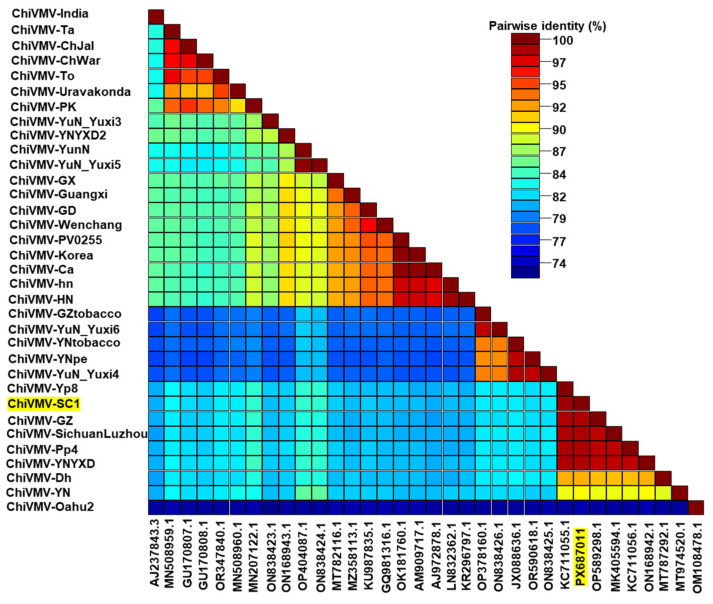
Pairwise identity of the complete genome sequences of different ChiVMV isolates analyzed using the Sequence Demarcation Tool version 1.2. The ChiVMV-SC1 isolate obtained in this study is highlighted.

To elucidate the relationship of the identified SC1 isolate to other ChiVMV isolates, a pairwise sequence identity analysis was conducted using the Sequence Demarcation Tool (SDT, version 1.2). The complete genome sequence of SC1 was compared with those of the other 34 ChiVMV isolates retrieved from the GenBank database. The pairwise sequence identities between SC1 and the other ChiVMV isolates ranged from 74.7% to 99.0% (Figure 2 and Table S2). The SC1 isolate showed the highest sequence identity (99.0%) with the Yp8 isolate (GenBank accession number KC711055.1) and the lowest identity (74.7%) with the Oahu2 isolate (GenBank accession number OM108478.1).

**Figure 3:**
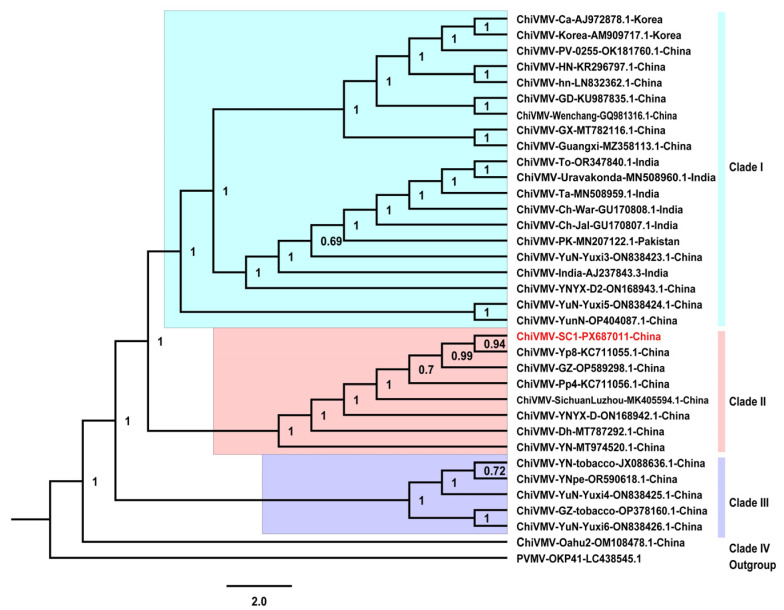
Phylogenies of the complete genome sequences of 35 ChiVMV isolates. The complete genome sequence of pepper veinal mottle virus (PVMV) was used as the outgroup. The accession numbers of the ChiVMV isolates used for phylogenetic analyses are provided in Table S3. The ChiVMV-SC1 isolate obtained in this study is highlighted in red.

To evaluate the phylogenetic relationship of the SC1 isolate, a phylogenetic tree was constructed based on the complete genomes of SC1 and the 34 ChiVMV isolates, using Pepper veinal mottle virus (PVMV, GenBank accession number LC438545.1) as the outgroup (Table S3). As shown in Figure 3, the 35 ChiVMV isolates clustered into four clades. Clade I consisted of 20 isolates originating from China, India, Korea and Pakistan, which were isolated from hosts including *Solanum nigrum*, tomato, and pepper. Clade II comprised SC1 along with isolates Yp8, GZ, Sichuanluzhou, Pp4, YNYX-D, and Dh, which were collected from tomato, pepper, or tobacco in China. Clade III contained isolates collected from tobacco and *S. nigrum* in China. Clade IV included only the Oahu2 isolate from the USA, indicating that it is distantly related to the other ChiVMV isolates (Table S3). These findings suggest that while geographical factors contribute to genetic divergence of ChiVMV isolates, multiple genetic lineages can coexist within the same geographical area. Furthermore, the potential diversity of host genotypes at the collection sites may also influence the phylogenetic clustering patterns.

### 2.3. Recombination Analysis

**Figure 4:**
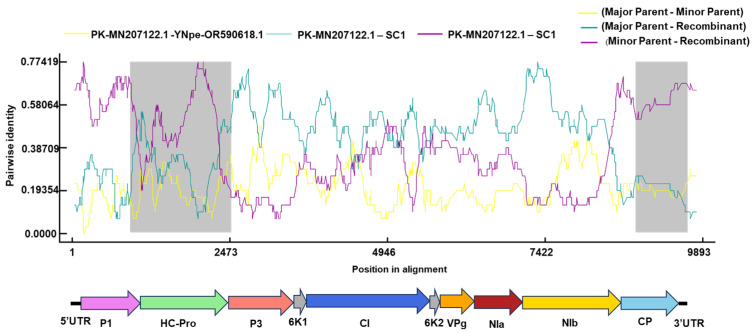
Recombinant analysis of the ChiVMV-SC1 isolate. The recombinant event was detected using the Recombination Detection Program RDP4. The blue and purple lines indicate the pairwise identities between the major parent (PK, MN207122.1) and the minor parent (YNpe, OR590618.1) and SC1, respectively. The gray regions denote the location of predicted breakpoints. The genomic organization of ChiVMV was indicated.

To understand whether recombination drives the divergence of ChiVMV, potential recombination events of ChiVMV were analyzed using the RDP program. 23 recombinant events were identified, with 28 isolates predicted to originate from recombination (Table S4). The analysis indicated that the SC1 isolate may derive from recombination, with the PK isolate (MN207122.1) as the putative major parent and the YNpe isolate (accession number OR590618.1) as the putative minor parent (Figure 4). This recombination event was detected with a high degree of confidence by seven recombination detection methods: RDP (average *p* value, 2.48 × 10^−36^), GENECONV (average *p* value, 3.16 × 10^−16^), Bootscan (average *p* value, 4.77 × 10^−39^), MaxChi (average *p* value, 1.27 × 10^−20^), Chimera (average *p* value, 3.57 × 10^−18^), SiSscan (average *p* value, 7.56 × 10^−29^) and 3Seq (average *p* value, 1.20 × 10^−12^).

### 2.4. Infectivity of the Full-Length cDNA Clone of ChiVMV

**Figure 5:**
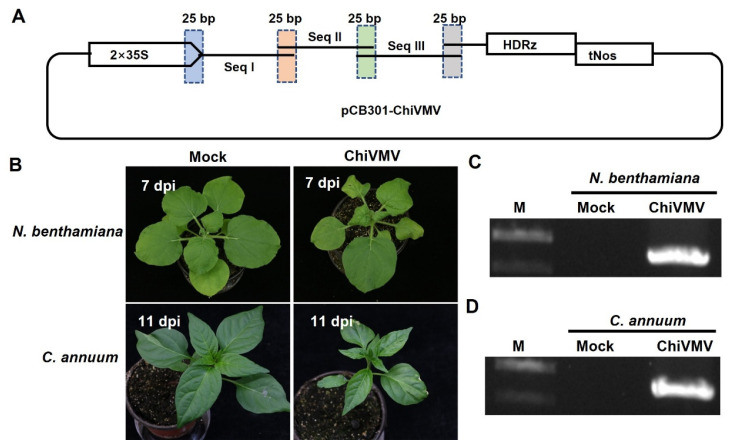
Infectivity of ChiVMV in *Nicotiana benthamiana* and *Capsicum annuum*. (**A**) Schematic diagram illustrating the one-step assembly cloning strategy used for the construction of the full-length cDNA clone of ChiVMV. Three overlapping ChiVMV fragments (Seq I, Seq II, and Seq III) were amplified by polymerase chain reaction and infused into the linearized pCB301 vector. 35S, the cauliflower mosaic virus promoter; HDRz, the hepatitis delta virus ribozyme; and tNos, the Nos terminator. (**B**) Symptoms of *N. benthamiana* inoculated with the infectious clone of ChiVMV at 7 days post inoculation (dpi) and *C. annuum* inoculated with ChiVMV-sap at 11 dpi. (**C**) RT-PCR analysis of ChiVMV RNA accumulation in systemic leaves of inoculated *N. benthamiana* using ChiVMV-specific primers (Chi-CP-F/R, Table S1). Lane M, GeneRuler 1 kb DNA ladders (Thermo); lane Mock, plants inoculated with the empty pCB301 vector; lane ChiVMV, *N. benthamiana* plants agroinoculated with pCB301-ChiVMV at 7 dpi. (**D**) RT-PCR detection of ChiVMV in systemic leaves of rub-inoculated *C. annuum* using ChiVMV-specific primers. Lane M, GeneRuler 1 kb DNA ladders (Thermo); lane Mock, samples mock-inoculated with PBS buffer; lane ChiVMV, systemic leaf samples from *C. annuum* plants mechanically inoculated with ChiVMV-sap at 11 dpi.

To investigate the pathogenicity of the SC1 isolate, the full-length cDNA of the SC1 isolate was inserted between the double 35S promoter and hepatitis delta virus ribozyme of the pCB301 vector to generate the infectious clone pCB301-ChiVMV, which is suitable for agroinfection (Figure 5A). To enhance the infection efficacy of the infectious clones, a 20-nt poly (A) tail was added to the 3′ end of the ChiVMV genome as described [25]. The infectivity of pCB301-ChiVMV was initially evaluated in the model plant *N. benthamiana* via *Agrobacterium*-mediated infiltration assays. At 7 days post-inoculation (dpi), typical symptoms such as leaf curling began to appear in the non-inoculated systemic leaves of all the 15 inoculated *N. benthamiana* plants (Figure 5B). The presence of ChiVMV RNA in these symptomatic leaves was confirmed by RT-PCR (Figure 5C). In contrast, control plants inoculated with the empty pCB301 vector showed no obvious symptoms and tested negative for viral RNA accumulation (Figure 5C). The infectious clone of pCB301-ChiVMV was subsequently used for agroinfiltration of *C. annuum*. However, the inoculated leaves developed necrosis symptoms and later abscised, preventing the virus from establishing systemic infection. Therefore, sap was harvested from the systemic leaves of *N. benthamiana* plants agroinfiltrated with pCB301-ChiVMV at 7 dpi and used for mechanical inoculation of *C. annuum* plants. At 11 dpi, newly emerged systemic leaves of the mechanically inoculated *C. annuum* plants displayed obvious mosaic symptoms. Compared to the mock-inoculated control plants, infection by ChiVMV caused stunting in *C. annuum* (Figure 5B). RT-PCR analysis confirmed the accumulation of viral RNA in the symptomatic leaves of sap-inoculated *C. annuum* plants (Figure 5D). These results indicate that the full-length cDNA clone of ChiVMV could replicate and move in *N. benthamiana* plants. Moreover, it can serve as an effective inoculum for infecting *C. annuum*.

### 2.5. Infectivity of the Myc-Tagged ChiVMV Infectious Clone

**Figure 6:**
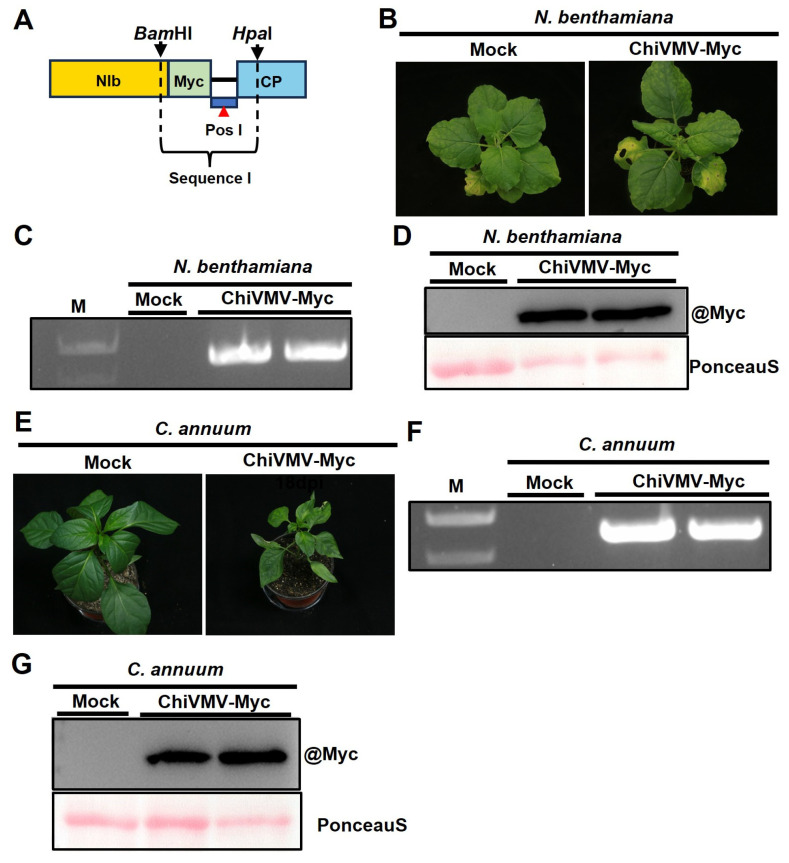
Infectivity of ChiVMV-NIb-Myc in *N. benthamiana* and *C. annuum*. (**A**) Schematic representation of Myc-tagged ChiVMV infectious clones. The Pos I indicates the introduced NIb cleavage recognition sequence. Sequence I was synthesized artificially, and the sequence is shown in Table S1. (**B**) Symptoms of *N. benthamiana* inoculated with the infectious clone of ChiVMV-NIb-Myc at 10 dpi. (**C**) RT-PCR analysis of ChiVMV RNA accumulation in systemic leaves of *N. benthamiana* inoculated with ChiVMV-NIb-Myc. (**D**) Western blot analysis of the expression of NIb-Myc in *N. benthamiana* samples inoculated with ChiVMV-NIb-Myc at 10 dpi. (**E**) Symptoms of *C. annuum* inoculated with the sap of ChiVMV-NIb-Myc-infected *N. benthamiana* plants at 18 dpi. (**F**) RT-PCR detection of ChiVMV in systemic leaves of rub-inoculated *C. annuum* using ChiVMV-specific primers. (**G**) Western blot analysis of the expression of NIb-Myc in *C. annuum* samples rub-inoculated with ChiVMV-NIb-Myc at 18 dpi.

Protein tags provide a practical alternative for the detection of viral proteins, especially when specific antibodies are unavailable. As NIb acts as a central hub that coordinates multiple processes critical for potyvirus infection, a Myc tag was inserted at the C-terminus of NIb. Unique restriction sites *Bam*HI in the NIb-encoding region and *Hpa*I in the CP-encoding regions were selected. The original sequences at these sites were replaced with synthetic sequences incorporating the Myc tag and preserving the native NIb cleavage site (Table S1), resulting in the successful construction of the recombinant infectious clone pCB301-ChiVMV-NIb-Myc (Figure 6A). Agroinfiltration of *N. benthamiana* with the recombinant clone pCB301-ChiVMV-NIb-Myc induced leaf curling symptoms indistinguishable from those induced by pCB301-ChiVMV (Figure 6B). RT-PCR analysis confirmed that viral RNA was present in the symptomatic systemic leaves (Figure 6C). Western blot analysis using an anti-Myc antibody showed that the NIb-Myc could be detected in these leaves (Figure 6D). These results suggest that the Myc insertion did not affect viral infectivity.

To assess its pathogenicity in the natural host, the sap was collected from the systemically infected *N. benthamiana* plants agroinfiltrated with pCB301-ChiVMV-NIb-Myc and mechanically inoculated onto *C. annuum* plants. At 18 dpi, the inoculated *C. annuum* plants exhibited mosaic and leaf curling symptoms (Figure 6E), consistent with those observed in *C. annuum* infected with pCB301-ChiVMV. Viral infection was confirmed by RT-PCR detection of viral RNA from the newly emerging systemic leaves of mechanically infected *C. annuum* (Figure 6F). Western blot analysis revealed the stable expression of the NIb-Myc fusion protein in these leaves (Figure 6G). The results indicate that the Myc-insertion was stable and did not affect the pathogenicity of ChiVMV.

## 3. Discussion

The significant impact of ChiVMV on global pepper production underscores the need to understand its epidemiology and pathogenicity [26,27]. In the present study, an isolate of ChiVMV (designated SC1) was identified from pepper plants collected in Sichuan, China, and its complete genome sequence was determined. Sequence analysis showed that the SC1 isolate shares the highest similarity (98–99%) with Yp8, Pp4, GZ and SichuanLuzhou isolates originating from southwest China. Despite being isolated from different hosts, the high sequence identity suggests that the genetic divergence of ChiVMV is closely associated with their geographic distribution. Consistent with this, phylogenetic analysis based on the complete genome sequence of ChiVMV isolates revealed that the 35 ChiVMV isolates clustered into four clades. The clustering pattern, wherein isolates from the same or geographically adjacent regions cluster together, further supports the role of geographic isolation in driving the genetic divergence of ChiVMV [28]. Notably, clade I contains ChiVMV isolates from China, India, Korea and Pakistan. Given that ChiVMV is transmitted by various aphid species in a non-persistent manner [15], it is plausible that aphid vectors with potential migratory ability have facilitated the cross-regional transmission of these ChiVMV isolates [29].

Recombination is a key factor driving virus evolution [30]. Recombination in geminiviruses enhances viral genetic diversity and facilitates rapid adaptation to the defense systems of different host plants [31,32]. Recombination of *Potyvirus* promotes the rapid emergence of new strains and enhances the transmission advantages of the viruses [33,34]. A previous study identified 10 recombination events among 25 ChiVMV isolates [28,35]. Here, analysis of 35 currently available complete genome sequences identified 23 putative recombination events, with the majority (28 out of 35) of ChiVMV genomes predicted to be recombinants. This prevalence indicates that recombination contributes more substantially to ChiVMV diversity than previously described [36,37]. The SC1 isolate characterized in this study was identified as a recombinant derived from a PK isolate from Pakistan and a YNpe isolate from Yunnan, China. The effect of recombination on different viruses varied. While recombination results in new, highly pathogenic strains of tomato yellow leaf curl virus and enhances its replication efficacy [38], the fitness of the new recombinant strain of watermelon mosaic virus is lower than that of the parent strains [36]. Although recombination is an important evolutionary force for potyviruses, the effect of recombination on the fitness and pathogenicity of ChiVMV warrants further investigation.

To understand the pathogenicity of the SC1 isolate, an infectious ChiVMV clone was constructed, and its infectivity and pathogenicity in *N. benthamiana* plants were verified. Moreover, sap harvested from infected *N. benthamiana* plants was mechanically transmissible and can induce typical symptoms in *C. annuum* plants. This is the first report of a ChiVMV infectious clone infecting pepper, providing a valuable tool for further studies of the function of viral proteins and virus–host interactions. It is noteworthy that *A. tumefaciens*-mediated infiltration of ChiVMV triggered a strong immune response in pepper leaves, leading to necrosis and abscission of the infiltrated leaves and prevention of viral infection. Recently, Liu et al. [39] obtained a mutant *A. tumefaciens* itex1 strain that induced a significant attenuation of the immune response and an increase in transient expression efficiency in pepper. Attenuated immune response may overcome this barrier and improve the infection efficiency of the ChiVMV clone in pepper.

Functional studies on ChiVMV-encoded proteins remain limited [40,41,42,43]. As the core protein of viral replication, NIb should theoretically play a key role in the interaction with the host, but the specific mechanisms by which ChiVMV NIb operates are poorly understood. Proteomics techniques based on antibody immunoaffinity of protein complexes and mass spectrometry have significantly accelerated unbiased analysis of virus–host protein interactions during viral infection [44]. The recombinant infectious clone ChiVMV-NIb-Myc in this study retained the pathogenicity in both *N. benthamiana* and pepper and stably expressed the NIb-Myc fusion protein. The availability of the ChiVMV-NIb-Myc clone developed here will enable identification of host proteins that interact with NIb, thereby providing insights into the functions of the NIb protein.

## 4. Materials and Methods

### 4.1. Plant Materials

*C. annuum* plants showing leaf curling and mosaic symptoms were collected from Chengdu City, Sichuan Province, China, in 2022. (Figure 1A). *C. annuum* seeds of the Xinxiang No. 8 cultivar were purchased from Jiangxi Nongwang High-tech. *C. annuum* and *N. benthamiana* plants were placed in a growth chamber at 25 °C with a 16:8 h (L:D) photoperiod.

### 4.2. Small RNA-Based Deep Sequencing

Total RNA was extracted from 1.0 g of mixed samples (pooled from three individual samples) of symptomatic *C. annuum* leaves using TRIzol reagent following the manufacturer’s protocol (Invitrogen, Carlsbad, CA, USA). The small RNA (sRNA) library was constructed using the Small RNA Sample Pre Kit (Illumina, San Diego, CA, USA). Following the ligation of the 3′ and 5′ adapters to sRNAs, the resulting sRNAs were reverse transcribed to complementary DNA (cDNA). The cDNA library was then PCR amplified and purified by PAGE. The resulting sRNA cDNA library was submitted to an Illumina HiSeq 2000 platform (Illumina, Inc., San Diego, CA, USA) for deep sequencing. After removing the adapter sequences and low-quality reads, sRNAs of 18–26 nt were assembled into contigs using Velvet 0.7.31 software with a k-mer size of 17 [45]. Candidate viral sequences were identified by querying the assembled contigs against the GenBank database by BLASTn on 12 May 2023.

### 4.3. Genome Assembly and Sequence Analysis

Three primer sets (Chi-1F/1R, Chi-2F/2R, Chi-3F/3R, Table S1) were designed to amplify overlapping regions covering the entire viral genome. First-strand cDNA was synthesized by reverse transcription of RNA extracted from symptomatic *C. annuum* leaves using the PrimeScript RT Reagent Kit (Takara, Tokyo, Japan). PCR amplification was conducted with the TransStart^®^ FastPfu DNA Polymerase (TransGen, Beijing, China). The amplified products were purified by a Gel Extraction Kit (Omega, Norcross, GA, USA) and ligated into a TA/Blunt-Zero Cloning Kit (Vazyme, Nanjing, China) for sequencing. The complete genome sequence of the virus was assembled by DNAStar 7.01 software (Madison, WI, USA) and deposited in the GenBank database under accession number PX687011.

### 4.4. Genome Characterization and Phylogenetic Analysis

Open reading frames (ORFs) encoded by the complete viral genome were predicted and annotated with reference to species belonging to the same genus. Sequence pairwise identity analysis was performed using Sequence Demarcation Tool version 1.2 (SDTv1.2) [46]. A phylogenetic tree was constructed based on the complete genome sequences of all ChiVMV isolates retrieved from the GenBank database, using the PVMV genome of the same genus as an outgroup. MAFFT v7.037 software [47] was used to align the complete genome sequences. The Sequence Matrix 1.7.8 program [48] was used to convert the aligned sequences into a NEXUS (non-interleaved) format file. The Bayesian inference (BI) method was used for phylogenetic analysis based on the combined gene dataset with the MrBayes 3.2.6 program [49]. The resulting phylogenetic trees were visualized and edited using FigTree v1.4.4 (https://github.com/rambaut/figtree/releases, accessed on 15 January 2026).

### 4.5. Plasmid Construction

The infectious clone of ChiVMV was constructed by homologous recombination using a ClonExpressII one-step cloning kit (Vazyme, Nanjing, China). Three overlapping DNA fragments were amplified using Chi-insert1F/1R, Chi-insert2F/2R and Chi-insert3F/3R (Table S1). PCR products were purified with a Gel Extraction Kit (Omega, Norcross, GA, USA). The pCB301 vector was linearized using the FastDigest restriction enzymes *Stu*I and *Sma*I (Thermo Fisher Scientific, Waltham, MA, USA), followed by homologous recombination reactions with three PCR fragments. The ligation products were transformed into *Escherichia coli* strain DH5α, and the recombinant plasmid of pCB301-ChiVMV was extracted and confirmed by Sanger sequencing.

Plasmids containing fragments (NIb partial sequence + Myc sequence + NIb cleavage recognition sequence + CP partial sequence, Table S1) were generated in Tsingke Biotechnology, and PCR products were obtained by amplification with Chi-myc-insert-F/R primers to generate Myc-tagged ChiVMV clones (Table S1). The products were purified and homologously recombined with the pCB301-ChiVMV plasmid linearized by *Bam*HI and *Hpa*I (Thermo Fisher Scientific, Waltham, MA, USA). The ligation products were transformed into the DH5α strain, and the recombinant plasmid of pCB301-ChiVMV-NIb-Myc was extracted and confirmed by Sanger sequencing.

### 4.6. Agroinfiltration of Plants

The recombinant plasmid pCB301-ChiVMV was transformed into *Agrobacterium tumefaciens* strain EHA105 via electroporation [50]. *A. tumefaciens* cells carrying each of the infectious clone constructs were incubated overnight at 28 °C. The cultures were centrifuged and resuspended with infiltration buffer containing 10 mM MgCl_2_, 10 mM MES (pH 5.6) and 100 mM acetosyringone to OD_600_ = 1.0. *A. tumefaciens* suspension was infiltrated into the abaxial side of two fully expanded leaves of 3- to 5-week-old tested plants using a needleless syringe. The inoculated plants were maintained in a growth chamber at 25 °C with a 16:8 h (L:D) photoperiod. Infiltration experiments were conducted three times, and five plants were used for each treatment.

### 4.7. Mechanical Inoculation

Systemically symptomatic leaves of *N. benthamiana* infiltrated with pCB301-ChiVMV or its derived vector were ground and used as inoculum. Approximately 1.0 g of symptomatic leaves was homogenized in 5 mL of 0.01 M PBS buffer (pH 7.2). For mechanical inoculation, two leaves per *C. annuum* plant were dusted with carborundum powder and gently rubbed with the prepared sap. Plants mechanically inoculated with PBS buffer were used as negative controls. After 5 min, the leaves were rinsed with tap water to remove excess inoculum. Plants were maintained in an insect-free greenhouse at 25 °C with a 16:8 h (L:D) photoperiod. Mechanical inoculation tests were conducted twice, and five plants were used for each treatment.

### 4.8. Protein Extraction and Western Blot Analysis

Total protein was extracted with the protein extraction buffer (containing 50 mM Tris-HCl (pH 6.8), 4.5% (*m*/*v*) SDS, 7.5% (*v*/*v*) 2-mercaptoethanol, 9 M carbamide). The protein extracts were denatured at 95 °C for 10 min, after which the supernatant was obtained by centrifugation at 12,000 rpm for 2 min. Protein samples were separated by 12% SDS-PAGE and then transferred to nitrocellulose membranes. Immunoblotting was performed with the primary antibody: mouse anti-Myc polyclonal antibody from Abcam (1:5000, cat. no. ab9106). After washing, the membranes were incubated with goat anti-mouse secondary antibodies obtained from Easybio (BE02, 1: 5000; Beijing, China). Blotted membranes were washed thoroughly, and the detection signal was visualized using an enhanced ECL chemiluminescent substrate kit (GE Healthcare, Chicago, IL, USA). Image results were generated using a chemiluminescence detection system (Tianneng, Shanghai, China).

## 5. Conclusions

In this study, the complete genome sequence of a ChiVMV isolate, designated SC1, was obtained from *C. annuum* in Sichuan Province, China. The SC1 isolate was identified as a recombinant, with the PK isolate from Pakistan as its major parent and a YNpe isolate from Yunnan, China, as its minor parent. A full-length infectious cDNA clone of ChiVMV was constructed and demonstrated to be infectious in *N. benthamiana* and host pepper. Furthermore, a recombinant clone with a Myc-tag inserted at the C-terminus of the NIb protein was generated for the first time. The derived infectious clone was still pathogenic and stably expressed the NIb-Myc fusion protein in infected plants. These results enhance the understanding of the genetic variability and evolution of ChiVMV and establish valuable reverse genetic tools for further studies of ChiVMV–host interactions.

## Data Availability

The original contributions presented in this study are included in the article/Supplementary Material. Further inquiries can be directed to the corresponding author.

## Supplementary Materials

The following supporting information can be downloaded at: https://www.mdpi.com/article/10.3390/ijms27021113/s1.
